# Canine Parvovirus and Its Non-Structural Gene 1 as Oncolytic Agents: Mechanism of Action and Induction of Anti-Tumor Immune Response

**DOI:** 10.3389/fonc.2021.648873

**Published:** 2021-05-03

**Authors:** Richa Arora, Waseem Akram Malla, Arpit Tyagi, Sonalika Mahajan, Basavaraj Sajjanar, Ashok Kumar Tiwari

**Affiliations:** ^1^ Division of Veterinary Biotechnology, ICAR-Indian Veterinary Research Institute, Izatnagar, India; ^2^ GB Pant University of Agriculture and Technology, Pantnagar, India; ^3^ Division of Biological Standardisation, ICAR-Indian Veterinary Research Institute, Izatnagar, India; ^4^ ICAR - Central Avian Research Institute, Izatnagar, India

**Keywords:** oncolytic virotherapy, apoptosis, canine parvovirus, nonstructural gene 1, anti-cancer agent

## Abstract

The exploration into the strategies for the prevention and treatment of cancer is far from complete. Apart from humans, cancer has gained considerable importance in animals because of increased awareness towards animal health and welfare. Current cancer treatment regimens are less specific towards tumor cells and end up harming normal healthy cells. Thus, a highly specific therapeutic strategy with minimal side effects is the need of the hour. Oncolytic viral gene therapy is one such specific approach to target cancer cells without affecting the normal cells of the body. Canine parvovirus (CPV) is an oncolytic virus that specifically targets and kills cancer cells by causing DNA damage, caspase activation, and mitochondrial damage. Non-structural gene 1 (NS1) of CPV, involved in viral DNA replication is a key mediator of cytotoxicity of CPV and can selectively cause tumor cell lysis. In this review, we discuss the oncolytic properties of Canine Parvovirus (CPV or CPV2), the structure of the NS1 protein, the mechanism of oncolytic action as well as role in inducing an antitumor immune response in different tumor models.

## Introduction

Advances in the field of modern medical sciences have not yet been able to prevent various types of cancers from wreaking havoc in terms of mortality and morbidity. One in every six deaths reported is due to cancer which makes this disease the second largest cause of death following heart ailments (1). The normal regulatory mechanisms that halt the overgrowth of cells and invasion of other tissues are disabled in tumor cells. Over time, cancer cells develop new features including modifications in cell structure, reduced cell adhesion, mutation, and production of new enzymes as the cells grow. These aberrations usually result from mutations in protein-coding genes that control cell division (2). Oncogenes and anti-apoptotic factors are usually activated whereas tumor suppressor genes are downregulated in cancer cells. Current cancer treatment modalities like chemotherapy, surgery, hormonal therapy, and immunotherapy alone or in combination, though reduce the impacts of a tumor but do not lead to a complete cure (3). Moreover, traditional treatment strategies have some severe side effects including non-specific action, narrow therapeutic index, drug resistance, and recurrence of cancer. These strategies are also reliant on the p53 gene’s functional status, which is mostly mutated in the vast majority of cancers, further diminishing the efficacy of such treatment methods (4). The utilization of numerous methods by neoplastic cells to escape efficient therapy can be the reason for the recurrence of the tumor (5). So, to fight the disease efficiently there is a continuous need to develop improved counter-strategies. The development of tumor cell specific therapeutic regimens, with no or minimal side-effects in combination with existing strategies that can be used to improve the quality of life for such patients, is a global concern.

Some viruses inhibit apoptosis for their multiplication, while several others do so to release their progeny (6). Several viruses selectively kill cancer cells while sparing normal cells. Such viruses, known as oncolytic viruses, have evolved various approaches that facilitate their existence and propagation in the host cells (7). Their viral capsid can act as a nano-sized nucleic acid delivery vehicle. Moreover, virus-induced effects are cell- and cell line-dependent, so the mechanism of inducing oncolysis will be different. Oncolytic viruses replicate inside cancer cells by taking advantage of the altered tumor micro-environment, inherent defects in anti-viral response, presence of tumor-specific receptors, and modified cellular pathways, ultimately killing them (8). T-Vec or Talimogene laherparepvec, a genetically modified Herpes virus is the first oncolytic virus (OV) approved by the FDA to treat melanoma in 2015 (9). Live viruses were first used for the treatment of cancer more than a century ago. The approach had to be soon abandoned due to viral toxicity, antiviral immunity, and re-emergence of viral virulence (10). However, advances in molecular biotechnology and genetic engineering that allow the usage of a particular cytotoxic viral gene to specifically target cancer cells have reignited the scientific interest in the development of viral gene-based cancer therapeutics. These viral genes are non-toxic, biodegradable, have no side effects, and target only malignant cells. Many other oncolytic viral genes are currently being investigated in clinical trials as candidates for cancer treatment, which includes E2 and E7 of Papillomaviruses, E1A 12S and 13S proteins, E3, and E4 of Adenovirus, SV40 large T antigen, tat of HIV-1, Parvovirus NS1, HN protein of Newcastle Disease Virus (NDV) and VP3 (apoptin) of Chicken Infectious Anemia Virus (CIAV) (11–18).

Viruses belonging to the genus Parvovirus have an intrinsic oncolytic property and cause cancer cell death. The gene responsible for the cytotoxicity of Parvovirus is the non-structural gene-1 (NS1). The oncolytic properties of various species of the Parvovirus genus have been thoroughly investigated (19, 20). Rat Parvovirus (H-1 PV) and Minute Virus of Mice (MVM) are well-known for their oncotropism and are efficient at infecting and destroying cancerous cells, while also suppressing tumor growth in animal models (21–23). Similarly, other parvoviruses, like Human Parvovirus (B19v) and Canine Parvovirus (CPV), are cytotoxic as well (24). These viruses kill cancer cells through a variety of mechanisms, including apoptosis, cytolysis, necrosis, and cathepsin-B-mediated cell death, depending on the cell type and development conditions (25–28). The oncolytic properties of Canine Parvovirus (CPV or CPV-2) will be discussed in this review, specifically concentrating on its NS1 protein structure, mechanism of action as well as its role in inducing an antitumor immune response.

## Parvovirus

**Figure 1:**
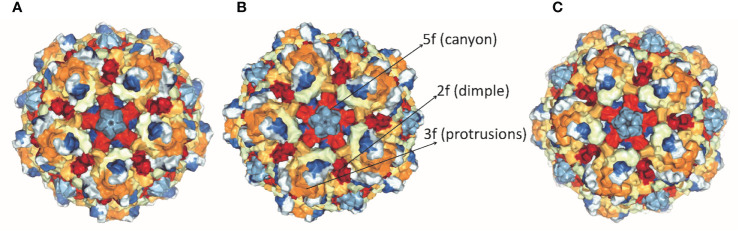
Structure of capsid of **(A)** H-1 PV, **(B)** MVM and **(C)** CPV. The capsids range in size from18 to 26 nm with icosahedral symmetry consisting of two, three, five-fold axes and have variable regions that affect virus-host cell interactions (PDB accession no.4gbt, 1z14, 2cas).

Parvoviruses have an overall diameter of ∼260 Å with T = 1 icosahedral capsid consisting of ∼5 kb linear ssDNA. Three capsid viral proteins (VP1, VP2, and VP3) and two non-structural proteins (NS1 and NS2) are encoded by the parvovirus genome, driven by promoters P4 and P38, respectively (19, 20). Both NS1 and NS2 proteins have a role in viral DNA replication but NS1 is the major mediator of cytotoxicity (29). Viral capsid proteins contain 60 copies of all three capsid proteins, with VP3 being the major component. Conserved amino acid sequence and similar post-translational processing of viral capsid proteins characterize rat virus-like (RV-like) subgroups of autonomous parvoviruses. CPV, MVM, and H-1 PV belong to this particular group (30). VP1 and VP3 proteins of MVM, H-1 PV, and CPV have a molecular mass of 83 and 64 kDa, 81 and 65 kDa, 82.3 and 65.7 kDa respectively (29–31). VP1 and VP2 are spliced from the same mRNA having common C-terminal amino acid sequences but VP1 has a unique N-terminal region. The structures of CPV, MVM, and H-1 PV capsids are approximately the same, except local surface conformational differences at three specific domains: (i) the icosahedral fivefold axis, (ii) icosahedral threefold axis, and (iii) icosahedral twofold axis, formed by anti-parallel beta strands and loops, as shown in **Figure 1**. VP3 is generated by cleavage from VP2 which exposes the conserved glycine-rich sequence through a five-fold axis. This conserved sequence is significant for the infectivity of parvoviruses as it binds with cellular membranes. VPs structure is conserved among parvoviruses, despite the difference in the amino acid sequence of family members. These differences mediate tumor tropism, receptor binding, and antibody recognition. For successful infection, members of the parvovirus genus, besides efficient capsid assembly, have evolved variable regions (VR0 to VR8) that contribute to local capsid surface variation of CPV, MVM, and H-1 PV for host-cell specific interactions (29, 31).

### Structure of NS1 Protein

**Figure 2:**
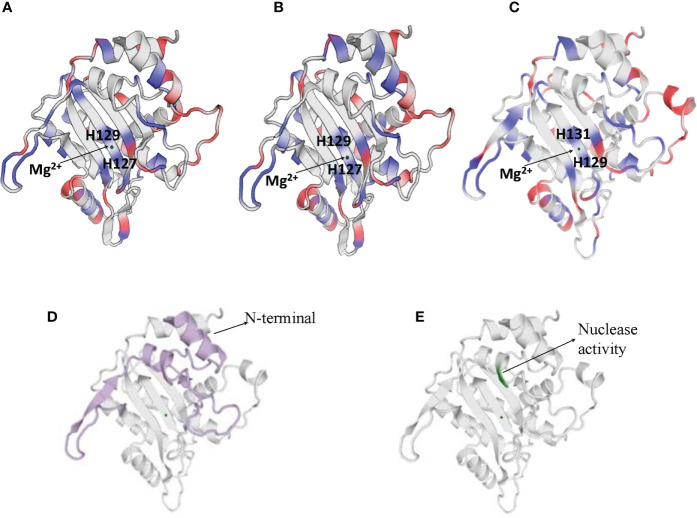
Cartoon presentation of N-terminal domain of NS1 protein of **(A)** H-1 PV (https://swissmodel.expasy.org/interactive/Yag5kS/models/), **(B)** MVM (https://swissmodel.expasy.org/templates/4r94.1) and **(C)** CPV (https://swissmodel.expasy.org/repository/uniprot/P12929). The catalytic DNA-nicking activity of viral replication proteins (nickase domain) is linked with the divalent metal ion coordination site, which comprises the conserved HUH motif (two histidines flanking a hydrophobic amino acid). **(D)** Colored portions depict N-terminal of CPV2.NS1 protein. **(E)** Green color represents the region with nuclease activity in CPV2.NS1 protein.

NS1 is a 76.7 kDa nuclear phosphoprotein, involved in several vital roles in the virus life cycle (32). It belongs to the superfamily 3 (SF3) helicases and contains an N-terminal DNA-binding/endonuclease domain (AA188–267), a central helicase domain (AA279–547), and a C-terminal zinc-finger domain (AA554–667) (33). NS1 proteins of CPV, H-1 PV, and MVM vary more in their N and C terminal sequences (as shown in **Supplementary Figure 1**), however, the helicase domains are predominantly conserved. NS1 protein sequence similarity percentages between CPV and MVM, CPV and H-1PV, MVM and H-1 PV are 72.62, 79.4, and 81.4% respectively. The proteins fold in an identical manner, as can be seen in **Figure 2**.

Numerous functions of NS1 are dependent on its association with DNA. N-terminal domain of NS1 identifies the viral ORI sequence and nicks the dsDNA genome in a site- and strand-specific manner. The formation of two ORI recognition complexes (OriL and OriR) occurs in the left and right-hand sides of the genome. NS1 along with dsDNA in Ori L binds with glucocorticoid modulatory element binding proteins, and in OriR binds with high-mobility group proteins forming ternary complex in both ORI (34, 35). At both of these recognition sites, nicking of the dsDNA genome occurs as a result of which NS1 is covalently linked to the newly formed 5′ end (36, 37). The catalytic DNA-nicking activity of viral replication proteins is linked with the divalent metal ion coordination site, which comprises a conserved HUH (two histidines flanking a hydrophobic amino acid) motif (38) and a linking tyrosine (37), as depicted in **Figure 2**. This HUH motif in CPV2.NS1 is formed by H129, H131 and E121 coordinated with a divalent metal ion and linking tyrosine which associates NS1 to 5′ end of the nicked DNA is Y212 (**Figure 2C**) (39). The N-terminal and the region with nuclease activity in CPV2.NS1 are depicted in **Figures 2D, E** respectively.

The helicase domain consists of a conserved ATP-binding pocket required for viral genome replication and is placed in the central part of the polypeptide chain (40). NS1 interacts with DNA in a sequence-independent manner but needs ssDNA overhang for initiation of replication (34, 41). Only the helicase function requires energy while ATP binding promotes NS1 oligomerization for other activities like recognition of ORI and nicking (34, 35, 40, 42, 43). Transactivation of viral capsid promoter P38 requires N and C termini of NS1, as a severe reduction in activation of P38 was reported in both N-terminal DNA recognition mutants and in C-terminal deletion mutants. Furthermore, it has been reported that NS1 protein intranuclear dynamics are largely dependent on its sequence-specific and nonspecific binding to double-stranded DNA, which relies on both the helicase domain’s conserved β-loop and the N-terminal domain (39, 44, 45)

## Canine Parvovirus and Its NS1 Gene as Potent Oncolytic Agents

Similar to other family members, CPV is a non-enveloped, linear, ssDNA virus with a 5 kb genome size and causes enteritis, leukopenia, and myocarditis in puppies. CPV uses transferrin receptors (TfR), which are highly expressed in neoplastic cells, to enter canine or feline cells. The virus has been known to bind to human TfR as well, but there is no evidence of CPV infecting humans (46). CPV2.NS1 performing multiple functions is required for a productive infection to take place. It can bind to specific DNA motifs, acts as a transcriptional activator, and is covalently attached to the 5’ end of the viral genome during DNA replication, where its nicking and helicase activities facilitate viral replication (37, 41, 47, 48). Because of its functions, NS1 is highly cytotoxic and reported to be mainly responsible for CPV-induced cell death.

### Oncotropism of CPV and NS1 in Transformed Cells

**Figure 3:**
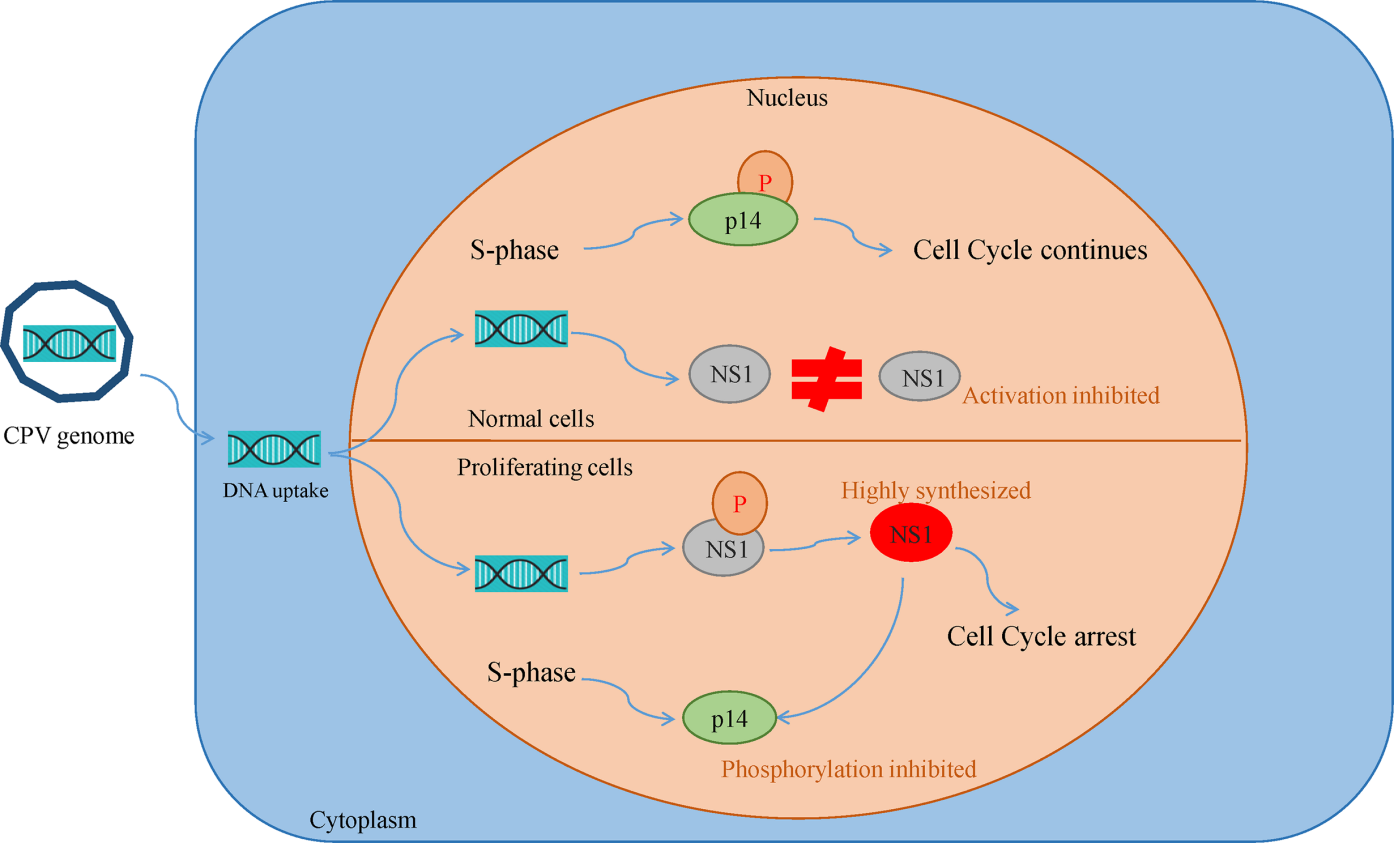
Oncotropism of CPV.NS1 in proliferating/transformed cells: CPV2.NS1 needs to be phosphorylated for activation. NS1 competes with a 14 kda protein (p14) for phosphorylation as both are phosphorylated at serine residues by the same protein kinase C family members. In normal cells, p14 undergoes phosphorylation, despite NS1 synthesis thereby preventing cell cycle arrest whereas, in proliferating cancer cells, NS1 synthesis occurs in huge amounts which inhibits p14 phosphorylation leading to cell cycle arrest and apoptosis.

Canine parvovirus lacks the mechanisms for inducing the S phase, thus it replicates only in proliferating host cells. Their inability to induce quiescent cells to proliferate is the reason for their oncotropism (49). This also implies that while most of the healthy cells are quite resistant to CPV cytotoxicity, they become sensitive as a consequence of their transformation. Cultures of normal rat fibroblast cell were observed to be resistant to NS1 (MVM, H-IPV) cytotoxicity as compared to transformed derivatives of the same cell. This effect may be caused by the existence of a greater fraction of cells with increased capacity for expression of viral mRNA and non-structural proteins. Another reason is NS1 interfering with the phosphorylation of a 14 kDa protein (p14) in the early S phase, which plays a role in DNA replication. Both NS1 and p14 are phosphorylated at serine residues during the same phase and so they might be the substrates of the same kinase. In normal cells, p14 phosphorylation occurs in S-phase despite NS1 synthesis but in transformed cultures due to high NS1 expression, it prevents p14 phosphorylation and competes for its own phosphorylation at serine residues (**Figure 3**). NS1 is also reported to decrease the level of 35 kDa (p35) protein which is similar to beta-tubulin, thus damaging the cytoskeleton and affecting cell motility, cell shape, and mitosis (50, 51).

## Mechanisms Involved in CPV and NS1 Mediated Cell Death

### DNA Damage and Cell Cycle Arrest

**Figure 4:**
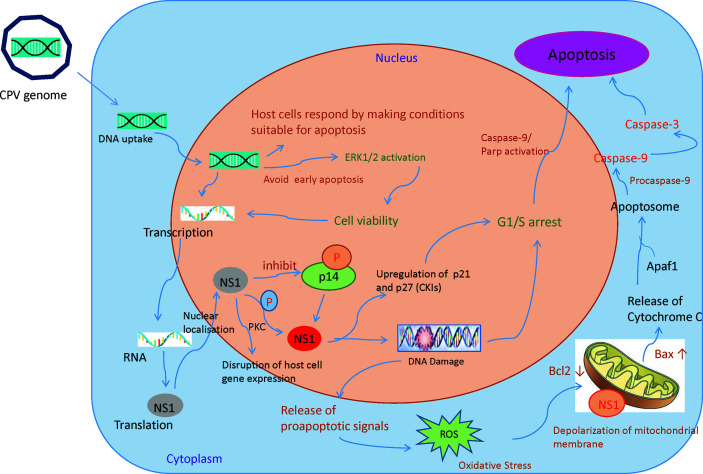
Mechanisms involved in CPV and NS1 mediated cancer cell death: Once the CPV genome is sensed by the host cells, they respond by activating caspase 9 and depolarizing the mitochondrial membrane. This causes the cells to undergo apoptosis. However, CPV activates cell survival pathways (ERK1/2) thereby mediating viral gene expression. CPV2.NS1 expression causes inhibition of host cell DNA replication. After activation by phosphorylation, NS1 causes DNA damage leading to cell cycle arrest at the G1/S phase before causing apoptosis. NS1 also associates with outer mitochondrial membrane and DNA damage response leads to activation of pro-apoptotic signals (Bcl2 and Bax) thereby creating oxidative stress. This cellular stress causes a decrease in mitochondrial membrane permeability, the release of Cytochrome C which further forms apoptosome complex with Apaf1, activating caspase 9, finally inducing cell death by intrinsic/mitochondrial pathway.

Recruitment of DNA damage response (DDR) kinases at the site of DNA damage causes silencing of cyclin-dependent kinases (CDKs) and cell cycle arrest, paving way for the removal of damaged cells through apoptosis (52). Depending on host cells and type of virus, Parvoviruses arrest cells at different phases (15, 53–57). CPV causes DNA disruption and interferes with the cell cycle, generating time to increase viral progenies leading to pathological consequences of infection, causing cell death after cell cycle arrest (**Figure 4**) (49). In the Norden Laboratory Feline Kidney cell line (NLFK) and the Canine Fibroma cell line (A72), CPV causes cell cycle arrest in the S phase (49, 58). Cell cycle arrest and DDR signaling evidently have significant role in canine parvovirus infections as inhibition of DDR interferes with viral replication and lowers the production of progeny (59). p53 expression is normally maintained at a very low concentration, however under certain conditions like DDR, hypoxia, and viral infection, p53 gene activation occurs which causes p21-mediated cell cycle arrest or apoptosis. CPV has been reported to activate p53 in Madin Darby Canine Kidney cells (MDCK) and Feline kidney cells, probably through up- and down-regulation of Bax and Bcl2 genes respectively (24, 60).

NS1 is an early gene product of CPV and its accumulation terminates host cell DNA replication. This results in a cell cycle arrest at S or G1 phase, followed by viral replication and an increase in NS1 gene expression. As NS1 is attached to DNA, it is presumed to be important in DNA encapsidation (61). NS1 protein’s various activities are regulated by protein kinase C family members phosphorylating serine residues in the protein. CPV2.NS1 like NS1 of MVM (56, 62, 63), H-1PV (15), and B19v (55), has been reported to induce DNA damage and cell cycle arrest in the G1 phase of the cell cycle (24, 64, 65). An increase in cyclin kinase inhibitors (CKIs) like p21 and p27 explains G1 arrest (66). CPV2.NS1 also causes phosphorylation of H2A.X, a common marker of DNA damage and DDR (64, 67). Internucleosomal DNA fragmentation is a hallmark of apoptosis reported to occur in CPV2.NS1 infected cells which is followed by Poly (ADP-ribose) polymerase 9 (PARP9) cleavage (64, 65). Caspase 3 causes the breakdown of this Poly (ADP-ribose) polymerase (PARP) into two fragments of 89- and 24-kDa that contains the DNA-binding domain and active site of the enzyme. This causes the inactivation of the PARP enzyme by hampering its capacity to react to DNA strand breaks and in this way PARP cleavage contributes to apoptosis (**Figure 4**). Unlike CPV, the CPV2.NS1 protein has been reported to cause apoptosis in a p53-independent manner. NS1 interaction with CREB binding protein (CBP) leads to interference with p53 transactivation, which could possibly explain why CPV2.NS1 causes apoptosis in HeLa and 4T1 induced mice mammary tumor cells in a p53-independent manner. Other Parvoviruses like MVM.NS1 also seem to cause apoptosis in the absence of p53 in some cells (68).

### Activation of Caspases

Caspases are proteases that are activated especially in the apoptotic cell death pathway (69). Caspase 8 is activated in death receptor-mediated apoptosis while caspase 9 activation is involved in the intrinsic/mitochondrial apoptotic pathway (70, 71). Caspase 9 is seen to be triggered early in a CPV infection whereas activation of caspase 8 has been detected during the later phase in NLFK and A72 cells (49). Activation/upregulation of caspase-8, caspase-9, and caspase-12 by CPV reported in MDCK cells indicates the involvement of extrinsic, intrinsic, and ER pathways (24). Further, there is the activation of caspase-3, the effector caspase, which confirms that the apoptosis is caspase-dependent (**Figure 4**). Caspase 8 is also activated in erythroid cells during B19v-induced apoptosis (72, 73).

CPV2.NS1 causes caspase 9 and 3 activation in both HeLa and 4T1 cells which indicates involvement of mitochondria in NS1 mediated cell death. Caspase 9 is also activated in various cell lines by the NS1 gene of other parvoviruses such as H-1PV, MVM, and B19v (15, 68, 74).

### Mitochondrial Involvement in CPV and NS1 Induced Oncolysis

Mitochondria determine the fate of cells from survival to death as they can sense intracellular stress through different signaling cascades and respond to those in order to get back to homeostasis (75). However, viruses modify mitochondrial function for their benefit (76). Changes in mitochondrial proteins and lipids, mtDNA mutations, mitochondrial transmembrane potential (DYm) depolarization, oxidative stress, and changes in mitochondrial number are some of the factors that cause mitochondrial dysfunction (77). As soon as the CPV is sensed by the host cells, the cells respond by making conditions for cells to undergo apoptosis by initially causing mitochondrial damage. However, it is observed that in NLFK and A72 cells, CPV infection causes activation of ERK1/2 cell survival pathway which restores mitochondrial function, preventing early apoptosis thereby promoting viral gene expression (**Figure 4**) (49).

The concentration of cytoplasmic calcium regulates mitochondrial membrane potential and increases during apoptosis as it is released from intracellular stores. CPV infection produces a small rise in cytoplasmic calcium concentration, implying that ER and mitochondrial calcium stores remain unchanged. In most cells, CPV causes apoptosis by interfering with the mitochondrial outer membrane. This leads to mitochondrial membrane depolarization due to apoptotic stimuli and proapoptotic signals (Bcl-2 family members like Bax and Bak) (78).

Bcl-2 and Bax regulate the integrity of mitochondrial membranes. CPV2.NS1 increases the BAX/Bcl2 ratio and initiates the destruction of the mitochondrial membrane potential (64, 79). Rise in oxidative stress by CPV2.NS1 infection in HeLa and 4T1 cells triggers aggregation of reactive oxygen species (ROS) resulting in the release of toxic substances like cytochrome C forming a multiprotein complex called “apoptosome” (69). But after treatment with an anti-oxidant agent, ROS production was observed to be reduced which suggested ROS may not be the sole mechanism for CPV2.NS1 mediated apoptosis (65, 80). However, ROS accumulation was reported to be the main cause of apoptosis in Porcine PV infected ST (swine testicles) cells (15, 79). Though, it is still uncertain how CPV2.NS1 can induce ROS accumulation, one of the possibilities could be deregulation of enzymes involved in ROS metabolism as CPV and its NS1 gene are closely associated with mitochondrial membrane (78). Similarly, the MVM, H-1 PV, and B19v NS1 proteins, also cause mitochondrial membrane depolarization and ROS aggregation in cancer cells (15, 68).

Proteomic changes in CPV-infected feline kidney cells also suggested that the mitotic cell cycle, apoptosis, the p53 gene, and mitochondria are involved in causing cell death, as major differentially expressed proteins linked to these pathways were found to be upregulated (60).

In CPV infected cells, cell death is mainly by apoptosis whereas, in the absence of phagocytes or when apoptotic cells are unable to eliminate them, secondary necrosis is also seen to occur (81, 82). On the other hand, H-1 PV infection has been reported to cause cell death by gathering of lysosomal cathepsins in cytoplasm and necrosis depending on specific cell types (28). However, MVM infection causes cell death by intervening with the cytoskeleton and modifying the substrate specificity of casein kinase II, which is different from typical apoptotic/necrotic cell death pathways (83, 84).

## Antitumor Immune Response of CPV2.NS1

**Figure 5:**
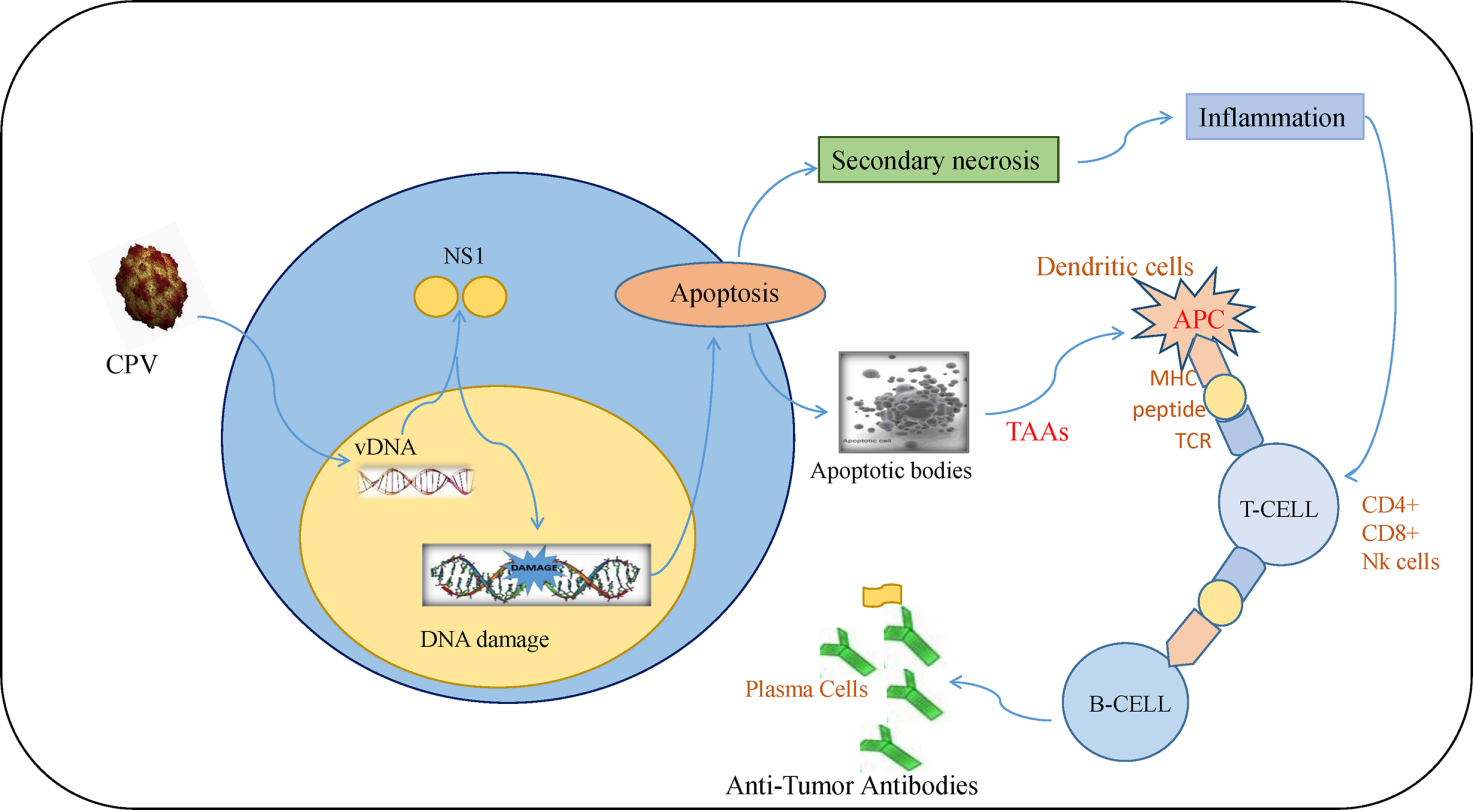
Immune response induced by CPV2.NS1 in cancer cells: CPV2.NS1 induces a potent anti-tumor immune response, thereby generating antibodies against tumor-associated antigens. Once CPV2.NS1 induces apoptosis or causes direct cell lysis, apoptotic bodies, and TAAs are released which are presented to MHC-I by antigen-presenting cells (APC). It also activates tumor-specific CTLs (CD8+ cells and Nk cells) which are involved in killing cancer cells. T-helper cells are also activated which help in maintaining CTL numbers and present the antigens to B-cells leading to the production of anti-tumor antibodies.

Tumor initiation, proliferation, and its response to treatment depend on the tumor microenvironment (85). The major anti-tumor immune response occurs by activation of CD8+ cytotoxic T lymphocytes (CTLs) whereas CD4+ T cells help in maintaining CTL numbers and support CTLs to gain entry into tumors (86). Although there are different mechanisms of escaping immune cell recognition by cancer cells, the direct destruction of neoplastic cells causes the presentation of tumor-associated antigens to MHC I by antigen-presenting cells (87–89). Lysis of cancerous cells by NS1 releases TAAs (tumor-associated antigens) that cause activation of CTL response specific to TAAs which leads to identification and destruction of tumor cells. This response also causes cross-presentation of TAAs by dendritic cells (DC) and further activation of tumor-specific CTLs (90). At this stage, tumor cells are targeted in a generalized manner even without the forced expression of CPV2.NS1 (**Figure 5**). Such type of oncolytic viral gene therapy can also be used as a cancer vaccine (91).

Intratumoral injection of CPV2.NS1 inhibited tumor cells expansion as illustrated by the significantly reduced tumor volume in the CPV2.NS1-treated 4T1 mice mammary tumor and DMBA mediated skin tumors in a rat tumor model. Tumor inhibition was manifested by necrosis, dryness, blackening, and a decrease in tumor size. There was a considerable increase in CD8+ cells, CD4+ cells, and NK cell populations in CPV2.NS1 treated group generating a strong anti-tumor immune response (65, 92). Necrosis and inflammation are helpful in oncolytic virotherapy in tumor cells for immune system activation (93). CPV2.NS1 causes secondary necrosis with uncontrolled lysis of dying cells leading to inflammation by releasing cellular debris (82) (**Figure 3**).

## Combination Therapy

### Combination of CPV2.NS1 and Immune Adjuvants for the Targeted Killing of Tumor Cells

CPV2.NS1 has been shown to trigger the immune system through its potential to destroy cancer cells by different processes, including apoptosis, necrosis and cell lysis (26–28, 65, 94). But, NS1 protein-mediated immune cell stimulation is mild in nature (65, 92) and the addition of an adjuvant may enhance it. Further, many tumors are weakly immunogenic, necessitating to incorporate an adjuvant to stimulate potent anti-tumor T cell immune response. Treatment with CPV2.NS1 and poly (I:C) as adjuvant alone caused tumor growth inhibition, but treatment with the conjunction of both the agents in the 4T1 induced mice mammary tumor model resulted in the development of a potent immune response. Th1 cytokines (IFN- and IL-2) are highly upregulated in CPV2.NS1 treated group, signifying the induction of cell-mediated immune response (95). Poly (I:C) has been known to decrease Myeloid-derived suppressor cells (MDSCs) which are one of the main immunosuppressive factors in 4T1 tumor cells (96–98). Poly (I:C) has also been observed to have an antiangiogenic effect and induce direct malignant cell destruction by TLR3 signaling receptors which are highly expressed in the 4T1 cell line (99–101).

VP3 gene of CIAV and CPV2.NS1 combination was used for the treatment of canine transmissible venereal tumor (CTVT) which did not cause any untoward effect and led to tumor regression in 10 to 14 days of gene therapy. This combination although caused partial regression but stopped further tumor development (102). In another study, apoptosis caused by bicistronic gene construct of NS1 gene of CPV2 and VP3 gene of CIAV, was observed to be moderate and lesser than the extent of apoptosis caused by chemotherapeutic agent nanosomal paclitaxel in canine mammary tumor (CMT) (3).

## Conclusions and Prospects

Cancer recurrence is the primary cause of failure of conventional treatment therapies as there are different pathways dysregulated in cancer cells. A single strategy/drug can block one pathway but another pathway will remain activated so combination therapy can be somewhat beneficial to resolve this problem. A few combination therapies with CPV2.NS1 have been discussed in this review but this aspect needs to be further addressed with two or more other strategies or drugs that act through different mechanisms. The success of cancer therapy is determined by how the treatment destroy tumor cells and engages the immune system to respond to cancer cells. To enhance the role of the immune system in killing cancer cells, CPV2.NS1 combination with other different forms of immunotherapy is a particularly promising strategy. Mostly all chemotherapeutic agents fail to induce programmed cell death when there is no functional p53 in most human cancer cells (4), so p53- independent apoptosis caused by the NS1 gene of CPV2 can serve as a beneficial therapeutic approach in such cancers. Identification of different key molecules/pathways involved in CPV and its NS1 gene-mediated cell death, -like recognition of kinase causing phosphorylation of NS1 for its activation-, would reveal their oncolytic mechanisms. These studies may also reveal key modulators of cells (either activated or repressed) that could predict whether our viral gene treatment is effective or not. Further such studies may guide to improve NS1 treatment, by recognition of new drugs that also target these key modulators which can be utilized in combination with NS1 to have a synergistic effect. There is a scope of investigation for ensuring safer and targeted oncolysis. This can be possible by choosing an efficient delivery vehicle for NS1 like liposomes, viral vectors, cell-penetrating peptides, etc. Lentiviral vectors can be used for the delivery of CPV2.NS1 as these vectors ensure stable integration of gene in the genomic DNA of the host and have previously been reported to be more efficient in the delivery of VP3 gene of CIAV in canine mammary tumor (CMT) cells as compared to lipofection (103). Antitumor activity of B18R gene of vaccinia virus was reportedly increased when incorporated in oncolytic herpes simplex virus, so recombinant viral vectors can also be used for delivery of viral genes (104). These new findings may guide further in optimizing CPV and CPV2.NS1 based therapies by overcoming existing molecular limitations with high efficacy.

## Author Contributions

RA and AKT conceived the idea for the article and prepared the first draft of the manuscript. RA and WM searched the relevant literature and prepared the figures. AT helped in drafting the manuscript. BS and SM contributed by critically revising its contents. All authors contributed to the article and approved the submitted version.

## Funding

This study was supported in part by National Agricultural Innovation Project (Project Code C4/3001) and CAAST-ACLH, ICAR-IVRI.

## Conflict of Interest

The authors declare that the research was conducted in the absence of any commercial or financial relationships that could be construed as a potential conflict of interest.

## Supplementary Material

Supplementary Figure 1 Multiple Sequence alignment of the NS1 protein of CPV, H-1 PV, and MVM. The protein sequences are less similar at the terminal but the structure is almost conserved in the middle portion of polypeptides (helicase domain).
