# Supplementary material for: Canine Parvovirus and Its Non-Structural Gene 1 as Oncolytic Agents: Mechanism of Action and Induction of Anti-Tumor Immune Response
Source: Front Oncol. 2021 May 3;11:648873. doi: 10.3389/fonc.2021.648873 (PMC8127782; doi:10.3389/fonc.2021.648873)
Supplement: Supplementary Figure 1 — Multiple Sequence alignment of the NS1 protein of CPV, H-1 PV, and MVM. The protein sequences are less similar at the terminal but the structure is almost conserved in the middle portion of polypeptides (helicase domain). [file DataSheet_1.pdf]

```
Reference sequence (1): GQ421597.1
Identities normalised by aligned length.
Colored by: identity


                    cov    pid   1 [        .         .         .         .         :         .         .         .         .         1         .         . 12
1 GQ421597.1     100.0% 100.0%     -------------------------------------------------MSGNQYTEEVMEGVNWLKKHAENEAFSFVFKCDNVQLNGKDVRWNNYTKPIQNEELTSLIRGAQTAMDQTE
2 NC_038545.1     99.7%  78.8%     -------------------------------------------------MAGNQYSEEVLDGVNWLKDQSDTEAFSFVFKCENVQLNGKDINWNNYNKPLNNEELTSLIRGAETAMDQA-
3 J02275.1        99.7%  67.5%     MISGSGSLNQGAKRKWAWFKVYKQLLKSVTYLFFHSVSRDAQKESNQLTMAGNAYSDEVLGATNWLKEKSNQEVFSFVFKNENVQLNGKDIGWNSYKKELQEDELKSLQRGAETTWDQS-
  consensus/100%                   .................................................MuGNtYo-EVhtusNWLKcpuppEsFSFVFKs-NVQLNGKDltWNsYpK.lpp-ELpSL.RGApTshDQs.
  consensus/90%                    .................................................MuGNtYo-EVhtusNWLKcpuppEsFSFVFKs-NVQLNGKDltWNsYpK.lpp-ELpSL.RGApTshDQs.
  consensus/80%                    .................................................MuGNtYo-EVhtusNWLKcpuppEsFSFVFKs-NVQLNGKDltWNsYpK.lpp-ELpSL.RGApTshDQs.
  consensus/70%                    .................................................MuGNtYo-EVhtusNWLKcpuppEsFSFVFKs-NVQLNGKDltWNsYpK.lpp-ELpSL.RGApTshDQs.

                    cov    pid 121          .         .         :         .         .         .         .         2         .         .         .         . 24
1 GQ421597.1     100.0% 100.0%     EEEMDWESEVDSLAKKQVQTFDALIKKCLFEVFVSKNIEPNECVWFIQHEWGKDQGWHCHVLLHSKNLQQATGKWPRRQMNMYWSRWLVTLCSVNLTPTEKIKLREIAEDSEWVTILTYR
2 NC_038545.1     99.7%  78.8%     -EDMEWESELDQLTKKQVQIFDALVKKCLFEVILNKNVAPNDCGWFIQHEWGKDQGWHCHVLLHGKGLQQAMGKWFRRQMNVLWSRWLVTACSVNLSPAERIKLREIAEDSEWVTVLTYR
3 J02275.1        99.7%  67.5%     -EDMEWETTVDEMTKKQVFIFDSLVKKCLFEVLNTKNIFPGDVNWFVQHEWGKDQGWHCHVLIGGKDFSQAQGKWWRRQLNVYWSRWLVTACNVQLTPAERIKLREIAEDNEWVTLLTYK
  consensus/100%                   .E-M-WEoplDphsKKQV.hFDuLlKKCLFEVh.sKNl.Ps-ssWFlQHEWGKDQGWHCHVLltuKshpQA.GKW.RRQhNhhWSRWLVThCsVpLoPsE+IKLREIAEDsEWVTlLTY+
  consensus/90%                    .E-M-WEoplDphsKKQV.hFDuLlKKCLFEVh.sKNl.Ps-ssWFlQHEWGKDQGWHCHVLltuKshpQA.GKW.RRQhNhhWSRWLVThCsVpLoPsE+IKLREIAEDsEWVTlLTY+
  consensus/80%                    .E-M-WEoplDphsKKQV.hFDuLlKKCLFEVh.sKNl.Ps-ssWFlQHEWGKDQGWHCHVLltuKshpQA.GKW.RRQhNhhWSRWLVThCsVpLoPsE+IKLREIAEDsEWVTlLTY+
  consensus/70%                    .E-M-WEoplDphsKKQV.hFDuLlKKCLFEVh.sKNl.Ps-ssWFlQHEWGKDQGWHCHVLltuKshpQA.GKW.RRQhNhhWSRWLVThCsVpLoPsE+IKLREIAEDsEWVTlLTY+

                    cov    pid 241          :         .         .         .         .         3         .         .         .         .         :         . 30
1 GQ421597.1     100.0% 100.0%     HKQTKKDYVKMVHFGNMIAYYFLTKKKIVHM-TKESGYFLSTDSGWKFNFMKYQDRQIVSTLYTEQMKPETVETTATTAQETKRGRIQTKKEVSIKCTLRDLVSKRVTSPEDWMMLQPDS
2 NC_038545.1     99.7%  78.8%     HKQTKKDYVKLVHFGNMIAYYFMTKKKIASSPPRDSGYFLSTDSGWKFNFMKEADRHLVSTLYTDQMKPETVETTVTTAQETKRGRIQTKKEVSIKCTLRDLVAKRVTSPEDWMMQQPDS
3 J02275.1        99.7%  67.5%     HKQTKKDYTKCVLFGNMIAYYFLTKKKISTSPPRDGGYFLSSDSGWKTNFLKEGERHLVSKLYTDDMRPETVETTVTTAQETKRGRIQTKKEVSIKTTLKELVHKRVTSPEDWMMMQPDS
  consensus/100%                   HKQTKKDYsKhVhFGNMIAYYFhTKKKIsp..s+-uGYFLSoDSGWKhNFhK.t-RplVSpLYT-pM+PETVETTsTTAQETKRGRIQTKKEVSIKsTL+-LVtKRVTSPEDWMM.QPDS
  consensus/90%                    HKQTKKDYsKhVhFGNMIAYYFhTKKKIsp..s+-uGYFLSoDSGWKhNFhK.t-RplVSpLYT-pM+PETVETTsTTAQETKRGRIQTKKEVSIKsTL+-LVtKRVTSPEDWMM.QPDS
  consensus/80%                    HKQTKKDYsKhVhFGNMIAYYFhTKKKIsp..s+-uGYFLSoDSGWKhNFhK.t-RplVSpLYT-pM+PETVETTsTTAQETKRGRIQTKKEVSIKsTL+-LVtKRVTSPEDWMM.QPDS
  consensus/70%                    HKQTKKDYsKhVhFGNMIAYYFhTKKKIsp..s+-uGYFLSoDSGWKhNFhK.t-RplVSpLYT-pM+PETVETTsTTAQETKRGRIQTKKEVSIKsTL+-LVtKRVTSPEDWMM.QPDS

                    cov    pid 361          .         .         .         4         .         .         .         .         :         .         .         . 48
1 GQ421597.1     100.0% 100.0%     YIEMMAQPGGENLLKNTLEICTLTLARTKTAFELILEKADNTKLTNFDLANSRTCQIFRMHGWNWIKVCHAITCVLNRQGGKRNTVLFHGPASTGKSIIAQAIAQAVGNVGCYNAANVNF
2 NC_038545.1     99.7%  78.8%     YIEMMAQPGGENLLKNTLEICTLTLARTKTAFDLILEKAEPSKLTTFVLGDTRTCRIFAGHGWNYIKVCHAIACVLNRQGGKRNTVLFHGPASTGKSIIAQAIAQAVGNVGCYNAANVNF
3 J02275.1        99.7%  67.5%     YIEMMAQPGGENLLKNTLEICTLTLARTKTAFDLILEKAETSKLTNFSLPDTRTCRIFAFHGWNYVKVCHAICCVLNRQGGKRNTVLFHGPASTGKSIIAQAIAQAVGNVGCYNAANVNF
  consensus/100%                   YIEMMAQPGGENLLKNTLEICTLTLARTKTAF-LILEKA-soKLTsFsLssoRTCpIFthHGWNalKVCHAIsCVLNRQGGKRNTVLFHGPASTGKSIIAQAIAQAVGNVGCYNAANVNF
  consensus/90%                    YIEMMAQPGGENLLKNTLEICTLTLARTKTAF-LILEKA-soKLTsFsLssoRTCpIFthHGWNalKVCHAIsCVLNRQGGKRNTVLFHGPASTGKSIIAQAIAQAVGNVGCYNAANVNF
  consensus/80%                    YIEMMAQPGGENLLKNTLEICTLTLARTKTAF-LILEKA-soKLTsFsLssoRTCpIFthHGWNalKVCHAIsCVLNRQGGKRNTVLFHGPASTGKSIIAQAIAQAVGNVGCYNAANVNF
  consensus/70%                    YIEMMAQPGGENLLKNTLEICTLTLARTKTAF-LILEKA-soKLTsFsLssoRTCpIFthHGWNalKVCHAIsCVLNRQGGKRNTVLFHGPASTGKSIIAQAIAQAVGNVGCYNAANVNF

                    cov    pid 481          .         5         .         .         .         .         :         .         .         .         .         6 60
1 GQ421597.1     100.0% 100.0%     PFNDCTNKNLIWIEEAGNFGQQVNQFKAICSGQTIRIDQKGKGSKQIEPTPVIMITNENITIVRIGCEERPEHTQPIRDRMLNIKLVCKLPGDFGLVDKEEWPLICAWLVKHGFESTMAN
2 NC_038545.1     99.7%  78.8%     PFNDCTNKNLIWVEEAGNFGQQVNQFKAICSGQTIRIDQKGKGSKQIEPTPVIMTTNENITVVRIGCEERPEHTQPIRDRMLNIHLTNTLPGDFGLVEKQEWPLICAWLVKNGYQSTMAS
3 J02275.1        99.7%  67.5%     PFNDCTNKNLIWVEEAGNFGQQVNQFKAICSGQTIRIDQKGKGSKQIEPTPVIMTTNENITVVRIGCEERPEHTQPIRDRMLNIHLTHTLPGDFGLVDKNEWPMICAWLVKNGYQSTMAS
  consensus/100%                   PFNDCTNKNLIWlEEAGNFGQQVNQFKAICSGQTIRIDQKGKGSKQIEPTPVIMhTNENITlVRIGCEERPEHTQPIRDRMLNI+LsppLPGDFGLV-KpEWPhICAWLVKpGapSTMAs
  consensus/90%                    PFNDCTNKNLIWlEEAGNFGQQVNQFKAICSGQTIRIDQKGKGSKQIEPTPVIMhTNENITlVRIGCEERPEHTQPIRDRMLNI+LsppLPGDFGLV-KpEWPhICAWLVKpGapSTMAs
  consensus/80%                    PFNDCTNKNLIWlEEAGNFGQQVNQFKAICSGQTIRIDQKGKGSKQIEPTPVIMhTNENITlVRIGCEERPEHTQPIRDRMLNI+LsppLPGDFGLV-KpEWPhICAWLVKpGapSTMAs
  consensus/70%                    PFNDCTNKNLIWlEEAGNFGQQVNQFKAICSGQTIRIDQKGKGSKQIEPTPVIMhTNENITlVRIGCEERPEHTQPIRDRMLNI+LsppLPGDFGLV-KpEWPhICAWLVKpGapSTMAs

                    cov    pid 601          .         .         .         .         :         .         .         .         .         7         .         . 72
1 GQ421597.1     100.0% 100.0%     YTHHWGKVPEWDENWAEPKIQEGINSPGCKDLE-TQAASNPQSQDQVLTPLTPDVVDLALEPWSTPDTPIAETAKQQSN--QLGVTHKDVQASPTWSEIEADLRAIFTSEQLEEDFRDDL
2 NC_038545.1     99.7%  78.8%     YSHHWGKVPDWSENWAEPAMQTPINSIGSARSTSQTATSTPLSQNYALTPLASDLADLALEPWSTPNTPVAEPVTGSNTN-TGGRNSQTARASPTWSEIEADLRACFSQEQLESDFNEEL
3 J02275.1        99.7%  67.5%     YCAKWGKVPDWSENWAEPKVPTPINLLGSARSPFTTPKSTPLSQNYALTPLASDLEDLALEPWSTPNTPVAGTAETQNTGEAGSKACQDGQLSPTWSEIEEDLRACFGAEPLKKDFSEPL
  consensus/100%                   Yst+WGKVP-WsENWAEPth.psIN..Gstc...ppstSsP.SQs.sLTPLssDl.DLALEPWSTPsTPlAtssptpss..thuhsppssphSPTWSEIEtDLRAhFstE.LcpDFp-.L
  consensus/90%                    Yst+WGKVP-WsENWAEPth.psIN..Gstc...ppstSsP.SQs.sLTPLssDl.DLALEPWSTPsTPlAtssptpss..thuhsppssphSPTWSEIEtDLRAhFstE.LcpDFp-.L
  consensus/80%                    Yst+WGKVP-WsENWAEPth.psIN..Gstc...ppstSsP.SQs.sLTPLssDl.DLALEPWSTPsTPlAtssptpss..thuhsppssphSPTWSEIEtDLRAhFstE.LcpDFp-.L
  consensus/70%                    Yst+WGKVP-WsENWAEPth.psIN..Gstc...ppstSsP.SQs.sLTPLssDl.DLALEPWSTPsTPlAtssptpss..thuhsppssphSPTWSEIEtDLRAhFstE.LcpDFp-.L

                    cov    pid 721    ] 723
1 GQ421597.1     100.0% 100.0%     D--
2 NC_038545.1     99.7%  78.8%     TLD
3 J02275.1        99.7%  67.5%     NLD
  consensus/100%                   s..
  consensus/90%                    s..
  consensus/80%                    s..
  consensus/70%                    s..
```

MView 1.63, Copyright © 1997-2018 Nigel P. Brown
